# Multimodal imaging-based prediction of recurrence for unresectable HCC after downstage and resection-cohort study

**DOI:** 10.1097/JS9.0000000000001752

**Published:** 2024-06-04

**Authors:** Hanyu Jiang, Mengxuan Zuo, Wang Li, Shuiqing Zhuo, Peihong Wu, Chao An

**Affiliations:** a Department of Minimal invasive intervention, State Key Laboratory of Oncology in South China, Guangdong Provincial Clinical Research Center for Cancer, Sun Yat-sen University Cancer Center; b Department of Radiology, State Key Laboratory of Oncology in South China, Guangdong Provincial Clinical Research Center for Cancer, Sun Yat-sen University Cancer Center, Guangzhou, Guangdong; c Department of Radiology, Functional and Molecular Imaging Key Laboratory of Sichuan Province, West China Hospital, Sichuan University, Chengdu, Sichuan, People’s Republic of China

**Keywords:** downstaging therapy, hepatocellular carcinoma, multimodal imaging, prognosis, surgical resection

## Abstract

**Background:**

Surgical resection (SR) following transarterial chemoembolization (TACE)-based downstaging is a promising treatment for unresectable hepatocellular carcinoma (uHCC), and identification of patients at high-risk of postoperative recurrence may assist individualized treatment.

**Purpose:**

To develop and externally validate preoperative and postoperative prognostic models integrating multimodal CT and digital subtraction angiography features as well as clinico-therapeutic-pathological features for predicting disease-free survival (DFS) after TACE-based downstaging therapy.

**Materials and methods:**

From March 2008 to August 2022, 488 consecutive patients with Barcelona Clinic Liver Cancer (BCLC) A/B uHCC receiving TACE-based downstaging therapy and subsequent SR were included from four tertiary-care hospitals. All CT and digital subtraction angiography images were independently evaluated by two blinded radiologists. In the derivation cohort (*n*=390), the XGBoost algorithm was used for feature selection, and Cox regression analysis for developing nomograms for DFS (time from downstaging to postoperative recurrence or death). In the external testing cohort (*n*=98), model performances were compared with five major staging systems.

**Results:**

The preoperative nomogram included over three tumors [hazard ratio (HR), 1.42; *P*=0.003], intratumoral artery (HR, 1.38; *P*=0.006), TACE combined with tyrosine kinase inhibitor (HR, 0.46; *P*<0.001) and objective response to downstaging therapy (HR, 1.60; *P*<0.001); while the postoperative nomogram included over three tumors (HR, 1.43; *P*=0.013), intratumoral artery (HR, 1.38; *P*=0.020), TACE combined with tyrosine kinase inhibitor (HR, 0.48; *P*<0.001), objective response to downstaging therapy (HR, 1.69; *P*<0.001) and microvascular invasion (HR, 2.20; *P*<0.001). The testing dataset C-indexes of the preoperative (0.651) and postoperative (0.687) nomograms were higher than all five staging systems (0.472–0.542; all *P*<0.001). Two prognostically distinct risk strata were identified according to these nomograms (all *P*<0.001).

**Conclusion:**

Based on 488 patients receiving TACE-based downstaging therapy and subsequent SR for BCLC A/B uHCCs, the authors developed and externally validated two nomograms for predicting DFS, with superior performances than five major staging systems and effective survival stratification.

## Introduction

Highlights

- Among 488 patients receiving transarterial chemoembolization-based downstaging and subsequent resection for unresectable Barcelona Clinic Liver Cancer (BCLC) A/B hepatocellular carcinoma from four hospitals, we developed two nomograms integrating tumor number, intratumoral artery, objective response, combined tyrosine kinase inhibitor, and microvascular invasion for predicting disease-free survival.
- The testing C-indexes of the preoperative (0.651) and postoperative (0.687) nomograms were higher than five major staging systems (0.472–0.542; all *P*<0.001).
- Two risk strata with distinct disease-free and overall survival outcomes were identified (all *P*<0.001).

Primary liver cancer is the sixth most common malignancy and third leading cause of cancer-related death worldwide, and hepatocellular carcinoma (HCC) accounts for 75–86% of these cases^1–4^. However, over 70% of HCCs are diagnosed at the intermediate to advanced stages^5^. Recently, growing data suggest the use of transarterial chemoembolization (TACE), as encouraging downstaging treatment with profound and durable efficacy, converting a proportion of unresectable HCC (uHCC) into resectable tumors, opening windows for subsequent curative-intent treatment^6,7^.

Surgical resection (SR) following successful TACE-based downstaging therapy is increasingly regarded as an effective treatment for uHCC^8^, but its long-term survival benefit is compromised due to frequent postoperative recurrence (32–54% at 5 years)^9^. Patients at high-risk for recurrence after SR following successful downstage are potential candidates for more intensive surgical approaches (e.g. wider resection margins), postoperative adjuvant therapies, and individualized surveillance with closer intervals and more sensitive techniques (e.g. MRI/CT over ultrasound)^10–14^. Therefore, identification of these high-risk patients may aid in individualized treatment and improve survival.

Growing evidence suggest that histopathologic characteristics, such as microvascular invasion (MVI) and the macrotrabecular-massive subtype, are associated with increased postoperative recurrence^15–17^. However, histopathological examination is an invasive method, subject to sampling errors, and usually unavailable before SR because HCC can be diagnosed noninvasively^18^. Fortunately, increasing studies showed that imaging techniques could profile the aggressiveness and prognosis of HCC in a noninvasive and comprehensive manner. For example, rim arterial phase hyperenhancement, nonsmooth tumor margins, and intratumoral artery been associated with MVI^19–24^. Several imaging-based prediction models were also proposed for HCC prognostication^25,26^. However, such a noninvasive decision-making tool for patients with uHCC undergoing SR following successful TACE-based downstaging therapy has not been yet identified.

Therefore, this multicenter study aimed to develop and externally validate preoperative and postoperative prognostic models based on multimodal imaging [CT and digital subtraction angiography (DSA)] and clinico-therapeutic-pathological features for predicting disease-free survival (DFS) in patients with uHCC receiving TACE-based downstaging therapy and subsequent curative-intent SR.

## Materials and methods

This multicenter, retrospective, cohort study was approved by the institutional review boards at all participating centers with a waiver of the requirement to obtain written informed consent. The study is reported according to the Strengthening The Reporting Of Cohort Studies In Surgery (STROCSS) criteria^27^ (Supplemental Digital Content 1, http://links.lww.com/JS9/C714).

### Patients

**Figure 1:**
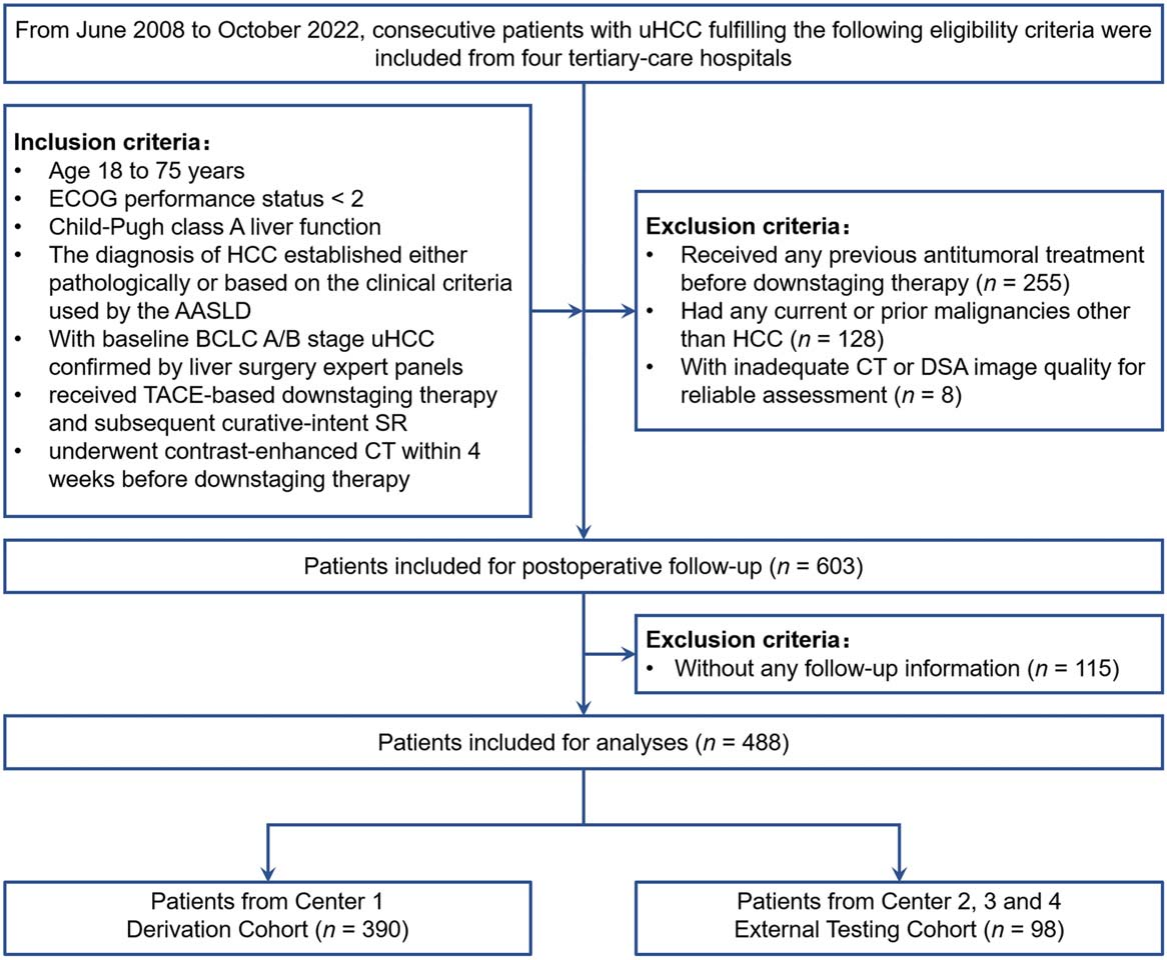
Study flowchart. TACE, transarterial chemoembolization; SR, surgical resection; HCC, hepatocellular carcinoma.

From June 2008 to October 2022, consecutive patients with uHCC were identified from four tertiary-care hospitals based on the following eligibility criteria. The inclusion criteria were: (a) age 18–75 years; (b) Eastern Cooperative Oncology Group (ECOG) performance status <2; (c) Child-Pugh class A liver function; (d) the diagnosis of HCC established either pathologically or based on the clinical criteria used by the American Association for the Study of Liver Disease^28^; (e) with baseline Barcelona Clinic Liver Cancer (BCLC)^28^ A/B stage uHCC confirmed by liver surgery expert panels (i.e. R0 resection was technically unachievable or remnant liver volume less than 30% in noncirrhotic patients or 40% in cirrhotic patients)^29^; (f) received TACE-based downstaging therapy and subsequent curative-intent SR; (g) underwent contrast-enhanced CT within 4 weeks before downstaging therapy. Patients were excluded if they: (a) received any previous antitumoral treatment before downstaging therapy; (b) had any current or prior malignancies other than HCC; (c) with inadequate CT or DSA image quality for reliable assessment; (d) without any follow-up information. Patient inclusion and exclusion are illustrated in Figure 1.

Clinical information (e.g. demographics, etiologies of chronic liver diseases) and laboratory results [e.g. alpha-fetoprotein (AFP) and platelet count] were collected at baseline as well as within 2 weeks prior to SR.

### Treatment

#### TACE-based downstaging therapy

TACE procedures were performed by three interventional radiologists (with 5–10 years of experience in TACE) at each participating center in a standardized manner according to institutional protocols. Whether to combine systemic therapy and the treatment regimens were determined based on discussions at the multidisciplinary tumor boards and personalized on the perceived probabilities of success and patient preference. TACE-based downstaging therapy details are provided in Supplementary Materials (E1.1) (Supplemental Digital Content 2, http://links.lww.com/JS9/C715).

Responses to TACE-based downstaging therapy was evaluated every 2–3 months after treatment initiation with the same imaging method as baseline according to the modified Response Evaluation Criteria in Solid Tumors (mRECIST) criteria^30^. Treatment responses were categorized as objective response (complete response or partial response) and nonobjective response (stable disease or progressive disease).

#### Surgical resection

SR would be recommended if tumor resectability (i.e. well-preserved liver function with the anticipation that R0 resection could be achieved successfully) was confirmed by liver surgery expert panels and objective response to downstaging therapy was achieved for at least 4 weeks. SR was performed by two surgeons (with 5–10 years of experience in liver surgery) at each participating center. Resection type (minimally-invasive or open surgery), extent (major or minor), and the application of anatomical resection were determined according to the surgeons’ discretion based on liver function, estimated residual liver volume, tumor burden, and comorbidities. Intraoperative ultrasonography was used routinely to evaluate minute intrahepatic tumors and the possibility of a negative resection margin. R0 resections was defined as tumor-free margin ≥1 mm for all detected tumors. All patients meeting the Chinese Society of Hepatology for hepatitis B virus (HBV) received anti-HBV treatment as clinically indicated^31^]. Postrecurrence treatments were discussed at the multidisciplinary tumor boards.

### Image analysis

The contrast-enhanced CT images before downstaging therapy and DSA images during TACE were reviewed independently by two fellowship-trained abdominal radiologists at each participating center who had 5–10 years of experiences in liver imaging. The reviewers were aware that all patients had HCC but were blinded to the remaining clinical, histopathologic, therapeutic, and follow-up information. All imaging features were evaluated on a per-patient basis. For patients with multiple tumors, the imaging features of the largest tumor were assessed.

**Figure 2:**
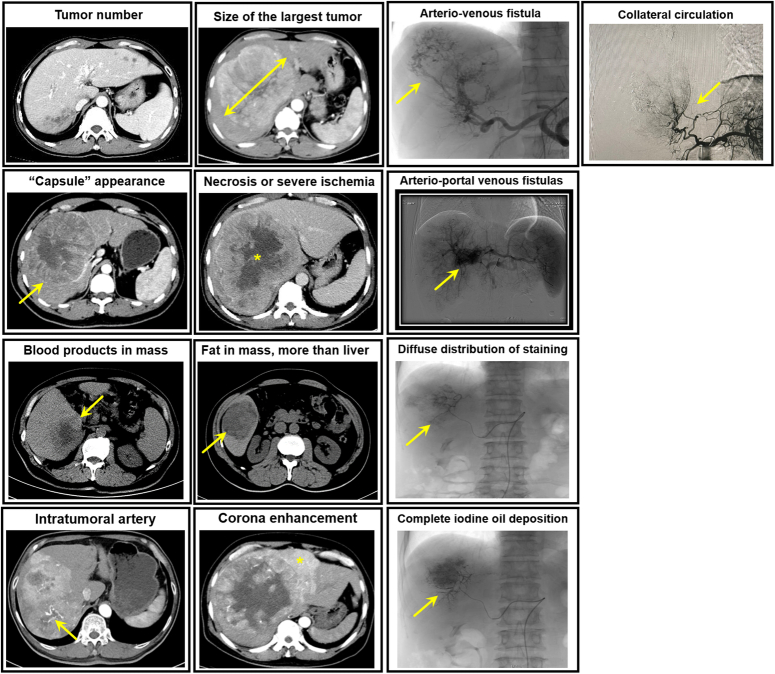
Examples of CT and digital subtraction angiography imaging features.

The following eight contrast-enhanced CT features were assessed: (a) tumor number (>three vs. ≤three), (b) size of the largest tumor (cm), (c) ‘capsule’ appearance, (d) necrosis or severe ischemia, (e) blood products in mass, (f) fat in mass, more than liver, (g) intratumoral artery, and (h) corona enhancement. The following five DSA features were assessed: (a) arterio-venous fistula, (b) arterio-portal venous fistula, (c) complete iodine oil deposition, (d) diffuse distribution of staining, and (e) collateral circulation. Imaging feature examples are shown in Figure 2, and their definitions summarized in Supplemental Materials (E1.2) (Supplemental Digital Content 2, http://links.lww.com/JS9/C715). For patients receiving multiple TACE procedures, the DSA features of the first TACE procedure were evaluated.

Inter-rater disagreements were resolved by a senior radiologist at each participating center who had over 20 years of experience in liver imaging.

### Histopathologic examination

The histopathologic data on satellite nodules and MVI were retrieved from routine pathology reports. According to the institutional standard operating procedures, all surgical specimens were examined by two pathologists (with 5–10 years of experience in liver pathology) at each participating center in consensus. For patients with multiple tumors, histopathologic data on the largest tumor were recorded.

### Follow-up and endpoints

All patients were regularly followed up after SR at 1 month and every 3–6 months thereafter with serum AFP and imaging techniques (contrast-enhanced ultrasound, CT or MRI) until death or the last follow-up date (31 October 2023).

DFS, defined as the time from the initiation of downstaging therapy to first-documented tumor recurrence (unequivocal radiological and/or histologic identification of HCC, tumor-in-vein, or distant metastasis as per American Association for the Study of Liver Diseases) or all-cause death, was regarded as the primary endpoint. Overall survival (OS), defined as the time from the initiation of downstaging therapy to death of any cause, was the secondary endpoint.

### Statistical analysis

Inter-rater agreement between the two reviewers was assessed with the intraclass correlation coefficient for tumor size and with Cohen’s Kappa value for binary imaging features. Patients from center 1 [*Sun Yat-sen University Cancer Center*] were assigned into the derivation cohort, while those from the remaining centers [*The First Affiliated Hospital of Sun Yat-sen University, Guangdong Provincial Hospital of Chinese Medicine and The Third Affiliated Hospital of Sun Yat-sen University*] into the external testing cohort. No formal sample-size calculation was performed beforehand, but the large number of derivation cohort DFS events compared to that of variables analyzed at the multivariable Cox regression analysis guaranteed the ‘10 events per variable’ rule of thumb, thus implying sufficient accuracy of the regression estimates^32^.

#### Development of the prognostic nomograms in the derivation cohort

Based on the derivation cohort, the XGBoost algorithm (version 1.6.1) was used for feature selection, and multivariable Cox regression analysis was performed for construction of the prognostic nomograms. Specifically, the XGBoost algorithm was applied to select the top important features associated with DFS among 40 multimodal imaging and clinico-therapeutic-pathological variables based on field knowledge (detailed in Supplementary Materials, E1.3, Supplemental Digital Content 2, http://links.lww.com/JS9/C715). The performance of the 40 evaluated variables for predicting DFS was evaluated with feature importance score according to the XGBoost algorithm. For each iteration, the three least important features (i.e. those with lowest feature importance scores) were removed, with the remaining features retrained in the XGBoost model. The above feature elimination process was repeated for 13 iterations (40/3 ~13) based on cross-validation to select the most important features. Variables selected by the XGBoost model are summarized in Supplementary Table 2 (Supplemental Digital Content 2, http://links.lww.com/JS9/C715) and illustrated in Supplementary Figure 1 (Supplemental Digital Content 2, http://links.lww.com/JS9/C715). Detailed parameters of the XGBoost model are presented in Supplementary Table 2 (Supplemental Digital Content 2, http://links.lww.com/JS9/C715). Computer codes for the XGBoost model are available in Supplementary Materials (E 1.4) (Supplemental Digital Content 2, http://links.lww.com/JS9/C715). The Shapley Additive exPlanations method was utilized to interpret the causal relationship of the XGBoost model^33^ (Supplementary Figure 2, Supplemental Digital Content 2, http://links.lww.com/JS9/C715). Thereafter, correlation and multicollinearity among the top important variables identified in the XGBoost model were assessed by Spearman’s correlation analysis and the variance inflation factor. In cases of collinearity (i.e. Spearman’s rho >0.6 or variance inflation factor >5), the strongest predictor was selected for further analyses. Finally, based on the selected features, multivariable Cox regression analyses with the backward stepwise method were performed to develop the prognostic nomograms. Two nomograms, namely the preoperative and postoperative nomograms, were respectively developed based on preoperative (clinical, imaging, and downstaging therapy-related) as well as postoperative (clinical, imaging, downstaging therapy-related, and histopathologic) variables.

To classify patients into high-risk and low-risk groups, the optimal thresholds of the nomograms were determined with X-tile software (version 3.6.1, Yale University School of Medicine, New Haven, Conn).

#### Validation of the prognostic nomograms and comparisons with major staging systems in the external testing cohort

Based on the external testing cohort, model discrimination was measured with the concordance index (C-index) and time-dependent area under the curve (td-AUC) from 12–60 months. Model calibration was evaluated by the calibration plot. Model prediction error was assessed by computing the integrated Brier score (IBS), and the clinical utility evaluated with decision curve analysis (DCA). The performances of the prognostic nomograms were compared with major clinical staging systems including the BCLC^34^, American Joint Committee on Cancer (AJCC)^28^, China Liver Cancer (CNLC)^4^, Japan Society of Hepatology (JSH)^35^, and Hong Kong Liver Cancer (HKLC) systems^36^ using Z test^37^.

#### Survival risk stratification

The cumulative survival outcomes of different risk groups were estimated using the Kaplan–Meier method and compared with the log-rank test, with subgroup analyses performed to adjust for known prognostic factors.

Statistical analysis was performed using SPSS version 23.0 (IBM Corp.) and the RMS package of R software version 3.5.1 (http://www.r-project.org/). All tests of significance were two-sided, and a *P*-value <0.05 was considered indicative of statistical significance.

## Results

### Patients

Baseline patient characteristics are summarized in Table 1. Briefly, 488 patients (age, 52.2±8.2 years; 426 men) were included, among whom 390 (age, 53.2±7.6 years; 341 men) from the derivation cohort, and 98 (age, 51.7±11.2 years; 88 men) from the external testing cohort. Chronic HBV infection was the predominant chronic liver disease etiology (94.5%, 461/488), and cirrhosis was detected in 455 (93.2%) patients.

**Table 1:** Patient characteristics.

	Derivation cohort	Testing cohort	
Characteristics	*n*=390	*n*=98	*P*
Clinical characteristics			
Age (years)	53.2±7.6	51.7±11.2	0.367^a^
Sex (male)	341 (87.4)	88 (89.8)	0.522^b^
BMI (kg/cm2)			
<18.4	46 (11.8)	5 (5.1)	0.150^b^
18.4–21.2	182 (46.7)	48 (49.5)	
>21.2	162 (41.5)	45 (45.9)	
Hepatitis B virus infection	370 (94.9)	91 (92.9)	0.625^b^
Portal pressure (kPa)	2.12±0.3	21.3±0.2	0.878^a^
Cirrhosis	366 (93.8)	89 (90.8)	0.846^b^
The Albumin-Bilirubin grade			
1	230 (59)	56 (57.1)	0.742^b^
2	160 (41)	42(42.9)	
The Barcelona Clinic Liver Cancer stage			
A	190 (48.7)	39 (39.8)	0.114^b^
B	200 (51.3)	59 (60.2)	
α-fetoprotein (>400 ng/ml)	178 (44.4)	46 (46.9)	0.646^b^
Albumin (g/l), mean±SD	39.9±7.8	38.5±8.9	0.762^a^
Aspartate aminotransferase (U/l), median, IQR	54 (17.2–118.3)	49 (15.3–122.7)	0.625^a^
Alanine aminotransferase (U/l), median, IQR	78 (22.5–256.8)	84 (31.6–287.2)	0.489^a^
Total bilirubin (μmol/l), mean±SD	11.2±2.2	9.8±2.0	0.556^a^
Platelet count (×10^9/l), median, IQR	226 (63–298)	268 (72–313)	0.382^a^
Neutrophils (ng/ml), mean±SD	4.5±1.7	4.4±1.2	0.988^a^
Lymphocyte (ng/ml), mean±SD	1.6±0.4	1.7±0.5	0.872^a^
Prothrombin time (s), mean±SD	11.2±2.2	11.0±1.8	0.818^a^
International normalized ratio, mean±SD	1.08±0.12	1.10±0.21	0.582^a^
C reactive protein (μmol/l), median, IQR	54.02 (9.11–113.26)	46 .23 (14.11–78.90)	0.675^a^
Creatinine (μmol/l), mean±SD	76.2±8.4	75.3±7.9	0.892^a^
Imaging characteristics			
CT features			
Tumor size (>7 cm)	237 (60.8)	55 (56.1)	0.402^b^
Tumor number (>three)	116 (29.7)	37 (37.8)	0.126^b^
‘Capsule’ appearance (present)	39 (10)	12 (12.2)	0.516^b^
Blood products in mass (present)	16 (4.1)	3 (3.1)	1.000^b^
Fat in mass, more than liver (present)	13 (3.3)	4 (4.1)	0.958^b^
Necrosis or severe ischemia (present)	61 (15.6)	19 (19.4)	0.370^b^
Corona enhancement (present)	104 (26.7)	24 (24.5)	0.661^b^
Intratumoral artery (present)	145 (37.2)	43 (43.9)	0.223^b^
DSA features			
Arteriovenous fistula (present)	54 (13.6)	19 (19.4)	0.169^b^
Arterio-portal venous fistula (present)	27 (6.9)	9 (9.2)	0.444^b^
Diffuse distribution of staining (present)	108 (27.7)	26 (26.5)	0.818^b^
Collateral circulation generation (present)	26 (6.7)	9 (9.2)	0.388^b^
Good iodine oil deposition (present)	90 (23.1)	26 (26.5)	0.473^b^
Histopathologic characteristics			
Microvascular invasion (present)	91 (23.3)	22(22.4)	0.853^b^
Satellite lesion (present)	44 (11.3)	20 (20.4)	0.017^b^
TACE parameters			
TACE sessions, median, IQR	2 (1–4)	2 (1–4)	1.000^a^
TACE exposure dose (Gy/cm2)	79.2 (43.4–102.2)	83.4 (54.9–112.7)	0.814^a^
TACE type			0.672^b^
cTACE	412 (66.6)	523 (63.7)	
dTACE	207 (33.4)	206 (36.3)	
Downstaging therapy characteristics			
TACE combined with tyrosine kinase inhibitors (yes)	127 (32.3)	34 (34.7)	0.654^a^
TACE combined with immune checkpoint inhibitors (yes)	92 (23.6)	22(22.4)	0.811^a^
Treatment response to downstaging therapy as per mRECIST			
Objective response (complete response or partial response)	216 (55.4)	50 (51.0)	0.438^a^
Nonobjective response (stable disease or progressive disease)	174 (44.6)	48 (49.0)	

Note.—Except where indicated, data are numbers of patients, with percentages in parentheses.

a Comparisons were made by the Student’s *t*-test or Mann–Whitney *U* test.

b Comparisons were made by the *χ*2 test or Fisher’s exact test.

Regarding TACE-based downstaging therapy, the median interval between downstaging therapy and SR was 4.5 months [interquartile range (IQR), 1.8–7.2 months] for the derivation cohort and 4.5 months (IQR, 1.8–7.2 months) for the testing cohort (*P*=0.999). The number of TACE procedure per patient was 3.5±0.8 for the derivation cohort and 3.2±0.5 for the testing cohort (*P*=0.807). A total of 153 (39.2%) derivation and 42 (42.9%) testing cohort patients received combined systemic therapy, respectively (detailed in Supplementary Table 1, Supplemental Digital Content 2, http://links.lww.com/JS9/C715). After downstaging therapy, the size of the largest tumor decreased from 7.2±1.3 cm to 3.5±1.1 cm (*P*<0.001) for the derivation cohort and from 7.0±1.5 cm to 3.3±0.8 cm (*P*<0.001) for the testing cohort. For adjuvant therapy, 36 (7.4%) patients received sorafenib, 49 (10.0%) received Lenvatinib, 31 (6.4%) received Apatinib, and 11 (2.3%) received Donafenib as the first-line treatment while 12 (2.5%) received Apatinib and 22 (4.5%) received Regorafenib as the second-line treatment.

During a median follow-up time of 32.2 months (IQR, 11.2–54.8 months), no difference in DFS was detected between the training and external testing cohorts (median DFS, 34.8 vs 34.2 months, *P*=0.985). The cumulative 1-year, 3-year, and 5-year DFS rates were 75.2, 51.6, and 37.5% for the derivation cohort, and 76.7, 53, and 36.6% for the testing cohort, respectively.

### Development of the prognostic nomograms in the derivation cohort

**Figure 3:**
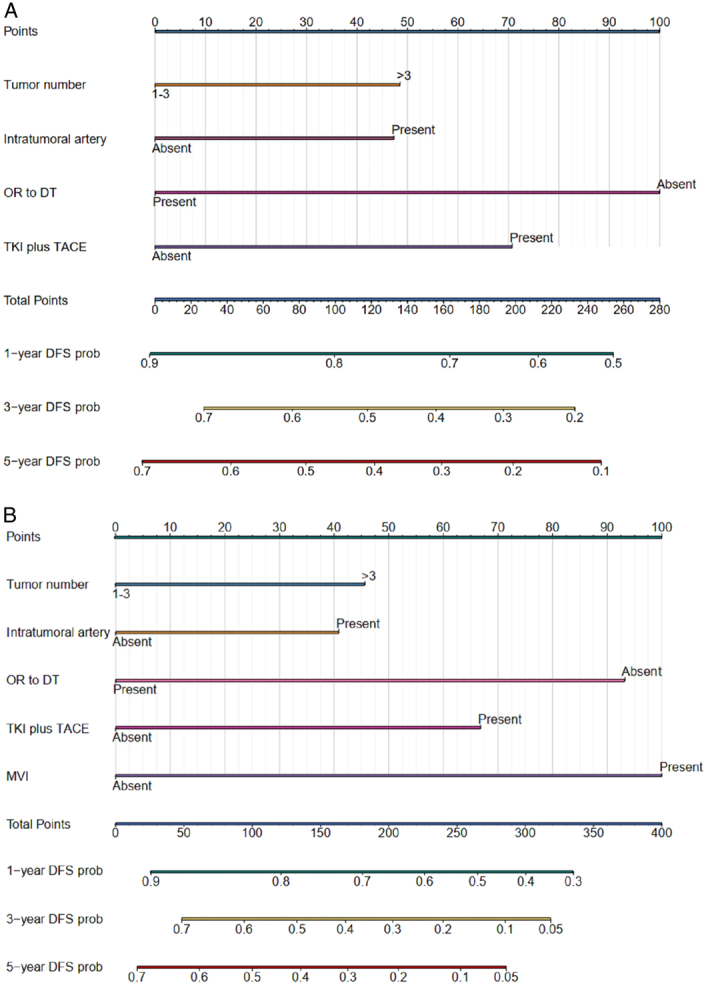
Preoperative (A) and postoperative (B) nomograms. DT, downstaging therapy; DFS, disease-free survival; MVI, microvascular invasion; OR, objective response as per modified Response Evaluation Criteria in Solid Tumors; TKI, tyrosine kinase inhibitor; TACE, transarterial chemoembolization.

Based on the derivation cohort, the XGBoost model identified 12 top important variables associated with DFS (Supplementary Table 2, Supplemental Digital Content 2, http://links.lww.com/JS9/C715, Figs. S1–2, Supplemental Digital Content 2, http://links.lww.com/JS9/C715). Detailed parameters of the XGBoost model are presented in Supplementary Table 3 (Supplemental Digital Content 2, http://links.lww.com/JS9/C715). Thereafter, the preoperative nomogram was developed based on tumor number (>three vs. ≤three) (HR, 1.42; 95% CI: 1.13–1.79; *P*=0.003), intratumoral artery (present vs. absent) (HR, 1.38; 95% CI: 1.09–1.76; *P*=0.006), TACE combined with tyrosine kinase inhibitor (TKI, yes vs. no) (HR, 0.46; 95% CI: 0.36–0.59; *P*<0.001) and objective response to down staging therapy (no vs. yes) (HR, 1.60; 95% CI: 1.26–2.04; *P*<0.001) (Fig. 3A). The postoperative nomogram was developed based on tumor number (> three vs. ≤three) (HR, 1.43; 95% CI: 1.08–1.90; *P*=0.013), intratumoral artery (present vs. absent) (HR, 1.38; 95% CI: 1.05–1.81; *P*=0.020), TACE combined with TKI (yes vs. no) (HR, 0.48; 95% CI: 0.36–0.64; *P*<0.001), objective response to downstaging therapy (no vs. yes) (HR, 1.69; 95% CI: 1.28–2.24; *P*<0.001) and MVI (present vs. absent) (HR, 2.20; 95% CI: 1.64–2.94; *P*<0.001) (Fig. 3B). The multivariable Cox analyses results are summarized in Table 2.

**Table 2:** Multivariable Cox regression analysis of predictors for disease-free survival following downstaging therapy based on preoperative and postoperative variables in the derivation cohort (*n*=390).

	Preoperative nomogram			Postoperative nomogram		
Variables	β	Hazard ratio	*P*	β	Hazard ratio	*P*
Tumor number (>three vs. ≤three)	0.350	1.42 (1.13–1.79)	0.003	0.358	1.43 (1.08–1.90)	0.013
Intratumoral artery (present vs. absent)	0.325	1.38 (1.09–1.76)	0.006	0.321	1.38 (1.05–1.81)	0.02
TACE combined with tyrosine kinase inhibitors (yes vs. no)	−0.770	0.46 (0.36–0.59)	<0.001	−0.732	0.48 (0.36–0.64)	<0.001
Objective response to downstaging therapy (no vs. yes)	0.472	1.60 (1.26–2.04)	<0.001	0.525	1.69 (1.28–2.24)	<0.001
Microvascular invasion (present vs. absent)	…	…	…	0.786	2.20 (1.64–2.94)	<0.001

Note.—Numbers in parentheses are the 95% confidence interval.

NA, not applicable; TACE, transarterial chemoembolization.

According to the preoperative nomogram, patients were stratified into either the high-risk (>68.72 points) or low-risk (≤68.72 points) groups. Similarly, according to the postoperative nomogram, patients were stratified into either the high-risk (>78.45 points) or low-risk (≤78.45 points) groups.

### Validation of the prognostic nomograms and comparisons with major staging systems in the external testing cohort

**Figure 4:**
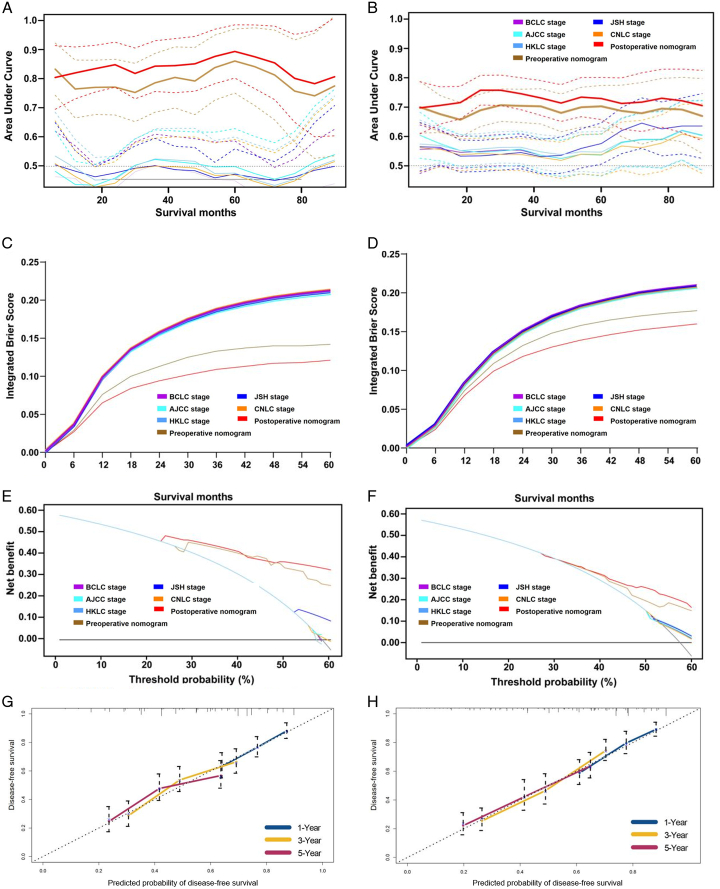
Performances of the preoperative and postoperative nomograms as well as five major staging systems for predicting disease-free survival. Model discrimination measured with time-dependent areas under the receiver operating characteristic curves at various time points for the derivation (A) and external testing (B) cohorts. Model overall fitness measured by integrated Brier score for the derivation (C) and external testing (D) cohorts. Model clinical usefulness measured by decision curves for the derivation (E) and external testing (F) cohorts. Model calibration measured by calibration plots for the preoperative nomogram (G) and postoperative nomogram (H) for the external testing cohort.

The discriminative abilities of the two nomograms and major staging systems are summarized in Table 3. Briefly, the testing cohort C-indexes of the preoperative (0.651) and postoperative nomograms (0.687) were higher than all five staging systems (C-index, 0.472–0.542; all *P*<0.001). Similarly, the testing cohort td-AUC of both the prenomograms and postnomograms were also higher than the other staging systems at various time points (all *P*<0.001) (Fig. 4A-B). The testing cohort IBSs for the preoperative and postoperative nomograms were 0.177 (95% CI: 0.113–0.253) and 0.160 (95% CI: 0.106–0.211), respectively (Fig. 4C-D). DCA demonstrated that both nomograms provided greater net benefit across the range of reasonable threshold probabilities than the staging systems in the testing cohort (Fig. 4E-F). Calibration curves plotted for 1-year, 3-year, and 5-year DFS show that both nomograms were well calibrated (Fig. 4G-H) in the testing cohort.

**Table 3:** Performances of the prognostic nomograms in comparison to five major staging systems.

	Derivation cohort (*n*=390)				External testing cohort (*n*=98)			
	Time-dependent				Time-dependent			
Models	C-index	*P* *	td-AUC†	IBS	C-index	*P* *	td-AUC†	IBS
Preoperative nomogram	0.734 (0.694–0.788)	0.004	0.802 (0.754–0.846)	0.142 (0.110–0.185)	0.651 (0.613–0.698)	0.021	0.701 (0.643–0.742)	0.177 (0.132–0.216)
Postoperative nomogram	0.779 (0.712–0.815)	Ref	0.840 (0.783–0.880)	0.121 (0.097–0.156)	0.687 (0.634–0.722)	Ref	0.728 (0.678–0.790)	0.160 (0.128–0.208)
BCLC	0.543 (0.498–0.608)	<0.001	0.540 (0.489–0.598)	0.206 (0.167–0.224)	0.522 (0.456–0.558)	<0.001	0.500 (0.452–0.547)	0.212 (0.178–0.256)
AJCC TNM	0.549 (0.501–0.612)	<0.001	0.542 (0.490–0.591)	0.205 (0.169–0.229)	0.472 (0.421–0.527)	<0.001	0.513 (0.461–0.587)	0.211 (0.166–0.254)
CNLC	0.537 (0.487–0.598)	<0.001	0.551 (0.502–0.611)	0.207 (0.172–0.234)	0.533 (0.473–0.532)	<0.001	0.410 (0.367–0.478)	0.211 (0.172–0.257)
HKLC	0.537 (4.582–0.615)	<0.001	0.537 (0.478–0.592)	0.207 (0.170–0.230)	0.533 (0.471–0.538)	<0.001	0.486 (0.412–0.524)	0.212 (0.173–0.255)
JSH	0.542 (0.489–0.611)	<0.001	0.552 (0.500–0.613)	0.206 (0.168–0.227)	0.542 (0.460–0.578)	<0.001	0.478 (0.436–0.527)	0.211 (0.170–0.245)

Note.—Numbers in parentheses are the 95% confidence interval.

* The *P* values were obtained from analyses comparing the C-indexes against the postoperative nomogram with the ‘survcomp’ package in R software.

† The time-dependent AUC represents the median td-AUC at various time points.

AJCC, American Joint Committee on Cancer; BCLC, Barcelona Clinic Liver Cancer; CNLC, China Liver Cancer; HKLC, Hong Kong Liver Cancer; IBS, integrated Brier score; JSH, Japan Society of Hepatology; td-AUC, time-dependent area under the receiver operating characteristic curve.

### Survival risk stratification

DFS outcomes according to risk groups defined by the preoperative and postoperative nomograms are summarized in Table 4.

**Table 4:** Disease-free survival outcomes according to risk groups defined by the preoperative and postoperative nomograms.

		Cumulative disease-free survival rate			
Risk groups	Disease-free survival (months)	2-year	5-year	Hazard ratio	*P* *
Preoperative nomogram					
Derivation cohort (*n*=390)					
Low- risk group	86.0 (23.2–104.8)	72.40%	56.70%	Ref	<0.001
High- risk group	20.2 (9.8–38.9)	45.50%	27.90%	2.37 (1.80–3.13)	
External testing cohort (*n*=98)					
Low- risk group	77.4 (18.7–97.5)	76.80%	60.50%	Ref	<0.001
High- risk group	15.8 (5.6–28.4)	43.10%	18.90%	3.28 (1.86–5.79)	
Postoperative nomogram					
Derivation cohort (*n*=390)					
Low- risk group	65.0 (18.7–93.8)	77.80%	56.80%	Ref	<0.001
High- risk group	18.0 (6.7–31.2)	35.50%	25.10%	2.94 (2.23–3.87)	
External testing cohort (*n*=98)					
Low- risk group	81.3 (22.7–99.5)	84.00%	64.10%	Ref	<0.001
High- risk group	13.4 (4.4–21.7)	38.10%	18.40%	4.68 (2.60–8.42)	

* The P values were computed using the log-rank test.

Note.—Numbers in parentheses are the 95% CI.

**Figure 5:**
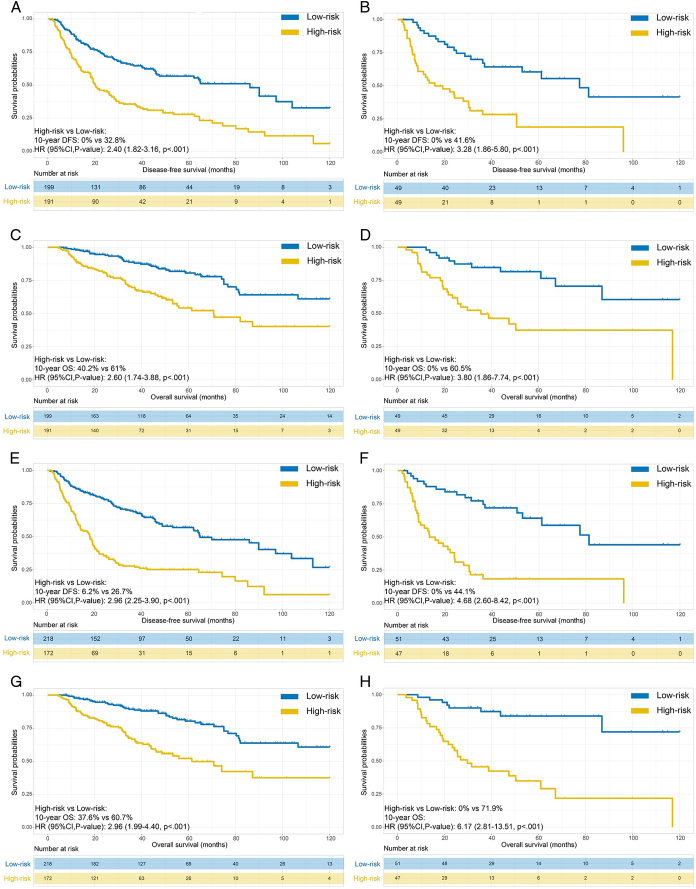
Kaplan–Meier curves for disease-free survival (DFS) and overall survival (OS) outcomes for different risk groups. DFS outcomes for risk groups defined by the preoperative nomogram for the derivation (A) and external testing (B) cohorts. OS outcomes for risk groups defined by the preoperative nomogram for the derivation (C) and external testing (D) cohorts. DFS outcomes for risk groups defined by the postoperative nomogram for the derivation (E) and external testing (F) cohorts. OS outcomes for risk groups defined by the postoperative nomogram for the derivation (G) and external testing (H) cohorts. DFS, disease-free survival; OS, overall survival.

According to the risk score threshold of the preoperative nomogram, the cumulative 5-year DFS rate of the low-risk group was higher than the high-risk group for both the derivation (56.7 vs. 27.9%, *P*<0.001) and testing (60.5 vs. 18.9%, *P*<0.001) cohorts (Fig. 5A-B). Likewise, according to the risk score threshold of the postoperative nomogram, the cumulative 5-year DFS rate of the low-risk group was also higher than the high-risk group for both the derivation (56.8 vs. 25.1%, *P*<0.001) and testing (64.1 vs. 18.4%, *P*<0.001) cohorts (Fig. 5C-D).

According to the preoperative nomogram, the cumulative 5-year OS rate of the low-risk group was higher than the high-risk group for both the derivation (84.2 vs. 34.8%, *P*<0.001) and testing (81.5 vs. 35.9%, *P*<0.001) cohorts (Fig. 5E-F). Likewise, according to the postoperative nomogram, the cumulative 5-year OS rate of the low-risk group was also higher than the high-risk group for both the derivation (84.6 vs. 35.2%, *P*<0.001) and testing (81.2 vs. 36.3%, *P*<0.001) cohorts (Fig. 5G-H).

**Figure 6:**
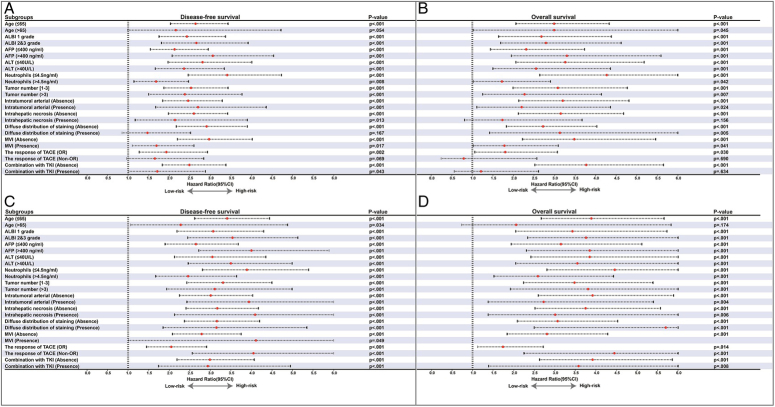
Subgroup analyses of disease-free survival (DFS) and overall survival (OS) between risk groups. (A) DFS comparisons between two risk groups based on the preoperative nomogram; (B) OS comparison between two risk groups based on the preoperative nomogram; (C) DFS comparison between two risk groups based on the postoperative nomogram; (D) OS comparison between two risk groups based on the postoperative nomogram. DFS, disease-free survival; OS, overall survival.

Better DFS and OS outcomes of the low-risk group than the high-risk group were observed across various subgroups (Fig. 6).

### Inter-rater agreement

The inter-rater agreement for tumor number was excellent for the derivation (Kappa, 0.963; 95% CI: 0.934–0.988) and testing (Kappa, 0.907; 95% CI: 0.854–0.964) cohorts. The agreement for intratumoral artery was also excellent for the derivation (Kappa, 0.825; 95% CI: 0.783–0.902) and testing (Kappa, 0.806; 95% CI: 0.759–0.876) cohorts.

## Discussion

SR following TACE-based downstaging therapy has been increasingly regarded as a safe and effective treatment option for patients with uHCC, but the long-term survival is limited by frequent postoperative recurrence^29^. Identifying patients at high-risk of recurrence could aid in individualized treatment-decision making. Therefore, based on 488 patients from four tertiary-care hospitals, we developed and externally validated two multimodal imaging-based nomograms for predicting DFS after downstaging therapy, which showed higher testing cohort C-index (all *P*<0.001), td-AUC (all *P*<0.001), lower prediction errors, and greater net benefit than five major staging systems.

By applying the XGBoost algorithm for feature importance ranking and mutual interference reduction, the top 12 risk factors most closely related to DFS were identified. In specific, the selected multimodal imaging features included tumor number, size of the largest tumor, and intratumoral artery on CT as well as arteriovenous fistula and diffuse distribution of staining on DSA^21,38,39^. To date, few prognostic DSA features have been identified in HCC, and this work, to our knowledge, represented the first attempt to combine the multimodal imaging features of CT and DSA for the prediction of HCC prognosis. Although no DSA feature was included in the final prediction nomograms, our results shed light on the potential of DSA features in prognostication of HCC, and future works are encouraged to further explore the prognostic utility and reproducibility of DSA features.

Two visual nomograms were respectively constructed based on preoperative and postoperative data. Specifically, tumor number, intratumoral artery, TACE combined with TKI, and objective response to downstaging therapy constituted the preoperative nomogram, and MVI was included in the postoperative nomogram in addition to the above preoperative features. Among them, tumor number and intratumoral artery were evaluated on pretreatment contrast-enhanced CT. The prognostic roles of tumor number in HCC patients receiving upfront SR^21^and those receiving liver transplantation after successful downstaging therapy^40^ have been well established, and our work further confirmed its utility in patients with uHCC receiving SR after TACE-based downstaging therapy. On the other hand, intratumoral artery has been increasingly regarded as a negative prognostic imaging feature in HCC and was correlated with more prominent angiogenesis, more frequent MVI^23,24,41^, and P53 mutation^42^.

Other than imaging features, two downstaging therapy-related variables, namely objective response to downstaging therapy and combination use of TKI were also included in the prediction nomograms. These results indicated that objective response as per mRECIST after downstaging therapy may inform the risk of recurrence after SR. Moreover, TKI combined with TACE was associated with better DFS in our work, and these results were in consistent with the recent global phase III randomized clinical trial (EMERALD-1), in which better progression-free survival was observed for the combination of bevacizumab, Durvalumab, and TACE over TACE monotherapy in patients with intermediate to advanced stage HCC. Similarly, another multicenter retrospective cohort study also revealed better progression-free survival of the combination of TACE plus programmed death-(ligand)1 inhibitors and molecular targeted treatments over TACE monotherapy in patients with intermediate HCC^43^. These findings underscored the potential synergistic antitumoral effects of TACE and antiangiogenic agents^44–46^. On the basis of tumor cell necrosis induced by TACE, the addition of TKI might further block tumor cell proliferation and promote apoptosis. Noteworthily, MVI was the most important risk factor associated with worse DFS among all variables included in the postoperative nomogram, which was consistent with the findings of previous reports^19–21^. This result highlights the paramount prognostic role of MVI in uHCC treated with TACE-based downstaging therapy.

Based on integrative analyses of multimodal imaging, clinical, therapeutic, and histopathologic features with rigorous feature selection and modeling methodologies, our prediction nomograms demonstrated superior prognostic performances than major staging systems in the external testing cohorts, which underscored their incremental values to current prognostic systems. The nomograms also allowed effective stratification of DFS as well as OS following downstaging therapy, which were stable across subgroup analyses when adjusting for known prognostic factors. These findings are clinically-relevant. First, objective response as per mRECIST was associated with better DFS in both the preoperative and postoperative nomograms. Therefore, treatment response might indicate the timing of SR after uHCCs being successfully downstaged within the resectability criteria. For example, continuous downstaging therapy might benefit patients with resectable tumors with mRECIST defined response of stable disease. Second, the preoperative nomogram may serve as a potential decision-making tool to inform personalized surgical planning. For instance, the high-risk patients may benefit from more intensive surgical approaches such as a wider resection margin. Third, the postoperative nomogram may be used to identify those high-risk patients who would be likely to benefit from postoperative adjuvant therapies as well as more intensive surveillance (e.g. shorter intervals and more sensitive techniques).

This study had several limitations. First, the retrospective design constituted an intrinsic limitation of the current study. Second, up to 96.1% of the included patients had chronic HBV infection, which might limit the extrapolations of our findings to non-HBV cohorts. Third, although we collected data from four tertiary-care hospitals, only 98 patients were included in the external testing cohort, but it is important to test the nomograms on a more diverse and larger population beyond the external testing cohort. Finally, due to the retrospective design and differences in routine practices, the TACE-based downstaging therapy regimens varied across the participating centers. These variations might have introduced confounding factors affecting our results. Therefore, future large scale prospective studies, ideally clinical trials, which enroll patients with more diverse chronic liver disease etiologies and standardized treatment regimens are warranted to validate and refine our findings.

In conclusion, based on 488 patients receiving TACE-based downstaging therapy and subsequent curative-intent SR for uHCC from four tertiary-care hospitals, we developed and externally validated two nomograms integrating multimodal imaging, clinical, therapeutic, and histopathologic features for predicting DFS after downstaging therapy. The nomograms showed higher predictive performances than five major staging systems and allowed effective stratification of DFS and OS risks, thus may aid in individualized treatment decision-making and surveillance strategy selection.

## Ethical approval

The study approved by the Institutional Review Board (or Ethics Committee) of Sun Yat-sen University Cancer Center (protocol code B2022-694-01).

## Consent

Written informed consent was obtained from the patient for publication of this case report and accompanying images.

## Source of funding

This work was supported by the National Natural Science Foundation of China (Grant No. 82101997) and the China Postdoctoral Science Foundation (Grant No. 2023T160448).

## Author contribution

P.H.W.: guarantors of integrity of entire study; C.A.: conception and design; H.J.Y.: development of methodology; W.L.: acquisition of data (provided animals, acquired and managed patients, provided facilities, etc.); M.X.Z.: analysis and interpretation of data (e.g. statistical analysis, biostatistics, and computational analysis); C.A.: writing, review, and/or revision of the manuscript; W.L.: administrative, technical, or material support (i.e. reporting or organizing data, constructing databases); P.H.W.: study supervision.

## Conflicts of interest disclosure

All authors have no conflicts of interest to disclose.

## Research registration unique identifying number (UIN)

Name of the registry: Researchregistry.com.

Unique identifying number or registration ID: researchregistry9425.

Hyperlink to your specific registration (must be publicly accessible and will be checked): https://www.researchregistry.com/.

## Guarantor

Peihong Wu is the guarantor.

## Data availability statement

The in-house developed medical database of this study is publicly accessible at http://www.yunedc.cn/#/login.

## Provenance and peer review

Not commissioned, externally peer-reviewed.

## Data availability statement

Data generated or analyzed during the study are available from the corresponding author by request.

## Supplementary Material
