# Supplementary material for: Multimodal imaging-based prediction of recurrence for unresectable HCC after downstage and resection-cohort study
Source: Int J Surg. 2024 Jun 4;110(9):5672–84. doi: 10.1097/JS9.0000000000001752 (PMC11392192; doi:10.1097/JS9.0000000000001752)
Supplement: Supplementary file 2 [file js9-110-5672-s002.docx]

**Multimodal Imaging-based Prediction of Recurrence for Unresectable HCC after Downstage and Resection: A Multicenter Study**

**Supplementary Materials**

1. **Supplementary methods and results**

E 1.1 TACE procedure details

E 1.2 Detailed imaging feature definitions

E 1.3 Clinical data collection

E 1.4 Computer codes for the XGBoost algorithm

1. **Supplementary Tables**

Supplemental Table 1. Systemic downstaging therapy regimens.

Supplementary Table 2. Variables Selected Using XGBoost and the Corresponding Variable Importance Score.

Supplementary Table 3. Parameters of the XGBoost model.

1. **Supplementary Figures**

Supplementary Figure 1. The 12 most important variables rank in XGBoost model.

Supplementary Figure 2. SHAP explain the nonlinear impact of the indictors variables on the early recurrence.

**1. Supplementary methods and results**

**E 1.1 TACE procedure details**

TACE procedures were performed by three interventional radiologists (with 5 to 10 years of experience in TACE) at each participating hospital in a standardized manner according to institutional protocols. Specifically, after accessing to the right femoral artery with an 18-gauge Seldinger needle, the celiac, hepatic, superior mesenteric artery was selectively catheterized, and digital subtraction angiography was acquired to assess the feeding arteries of tumors. The catheter was super-selectively placed into the feeding segmental or subsegmental arteries using selective hepatic angiography and/or tracking navigation imaging. Conventional TACE or drug-eluting-bead TACE was performed at the discretion of intervention radiologists. For conventional TACE, chemoembolization was performed using emulsion, which consisted of 10-20 ml lipiodol, 30-50 mg platinum drugs, and 20-40 mg epirubicin. Emulsion was injected slowly until the offending vessel occluded. Afterwards, embolization using gel foam mixed with contrast medium was injected to reduce the residual blood flow until there was no longer any tumor staining after repeat angiography. Drug-eluting-bead TACE was performed using drug-eluting beads loaded with doxorubicin in a super-selective manner. For patients with very large tumors or with insufficient liver preservation, split TACE was allowed with a 4-6 weeks interval. TACE procedure was repeated on demand every 6-8 weeks if follow-up images showed evident viable tumors.

**E 1.2 Detailed imaging feature definitions**

| **Imaging features** | **Definitions** |
| --- | --- |
| ***CT features*** | |
| Tumor numer (>three vs. ≤three) | Number of unequivocal HCC according to the clinical criteria used by the American Association for the Study of Liver Diseases. |
| Size of the largest tumor (cm) | Largest outer-edge-to-outer-edge dimension of the dominant tumor. |
| "Capsule" appearance (present vs. absent) | Smooth, uniform, sharp border around over 50% of a tumor, unequivocally thicker or more conspicuous than fibrotic tissue around background nodules. |
| Fat in mass, more than liver (present vs. absent) | Excess fat within a mass, in whole or in part, relative to the adjacent liver. |
| Necrosis or severe ischemia (present vs. absent) | Presence of nonenhancing area in a solid mass, not attributable to a cystic component, prior treatment, or intratumoral hemorrhage. |
| Blood products in mass (present vs. absent) | Intratumoral hemorrhage in the absence of biopsy, trauma, or intervention. |
| Corona enhancement (present vs. absent) | Peritumoral enhancement in the late arterial phase or early portal venous phase. The enhancement is contiguous with and surrounds all or part of the tumor. |
| Intratumoral artery (present vs. absent) | Presence of discrete arteries within the tumor on arterial phase images. |
| ***DSA features*** | |
| Arterio-venous fistula (present vs. absent) | Presence of abnormal connection between hepatic arteries and veins, allowing arterial blood to flow into the hepatic venous vessels. |
| Arterio-portal venous fistula (present vs. absent) | Presence of abnormal connection between hepatic arteries and portal veins, allowing arterial blood to flow into the portal venous vessels. |
| Complete iodine oil deposition (present vs. absent) | Presence of complete deposition of iodine oil within the tumoral area. |
| Diffuse distribution of staining (present vs. absent) | Presence of diffuse distribution of tumor staining. |
| Collateral circulation (present vs. absent) | Presence of collateral capillaries to supply the tumoral area despite occlusion of the major feeding vessels. |

**E1.3 Clinical data collection**

The following clinical variables were collected:

1. **Demographic and clinical variables**, including age, sex, body mass index, Eastern Cooperative Oncology Group performance status, comorbidities (i.e., hypertension, diabetes, heart disease, renal disease and esophageal gastric varices), etiology of chronic liver disease, Albumin-bilirubin grade, and ascites;
2. **Laboratory variables**, including α-fetoprotein, albumin, total bilirubin, aspartate aminotransferase, alanine aminotransferase, creatinine, C reactive protien, platelet counts, neutrophils, lymphocytes, prothrombin time, and international normalized ratio;
3. **Downstaging therapy-related variables**, including treatment response to downstaging therapy as per mRECIST, TACE combined with tyrosine kinase inhibitors, and TACE combined with immune checkpoint inhibitors;
4. **Tumor stage according to five major staging systems**, including the Barcelona Clinic Liver Cancer (BCLC), American Joint Committee on Cancer (AJCC), China Liver Cancer (CNLC), Japan Society of Hepatology (JSH), and Hong kong Liver Cancer (HKLC) systems.

**E1.4 Computer codes for the XGBoost model**

import numpy as np

import pandas as pd

from sklearn.model_selection import train_test_split

from sklearn.model_selection import KFold, cross_val_score as CVS

from sklearn.feature_selection import RFECV

import matplotlib.pyplot as plt

from xgboost import XGBClassifier

import seaborn as sns

import matplotlib.ticker as ticker

from sklearn.model_selection import StratifiedKFold

def miss_fillup(df, cols, method):

from sklearn.impute import SimpleImputer

for col in cols:

tmp = df.loc[:,col].values.reshape(-1,1)

imp = SimpleImputer(strategy=method).fit_transform(tmp)

df.loc[:,col] = imp

return df

def col_valcount(df, col):

return df[col].value_counts().sort_index()

from sklearn.metrics import accuracy_score, precision_score, recall_score, roc_auc_score, f1_score, roc_curve, auc, confusion_matrix

import matplotlib.pyplot as plt

import pandas as pd

import numpy as np

class EvaModelFusion:

def __init__(self):

pass

def train(self, X, y):

self.X_train = X

self.y_train = y

def cal_res(self, clf, X_test, y_test, output=False, sm = False):

clf.fit(self.X_train, self.y_train)

# y_test_preds = clf.predict(X_test)

if sm == True:

y_test_predprob = clf.decision_function(X_test)

else:

y_test_predprob = clf.predict_proba(X_test)[:,1]

# 计算约登指数, youden's index

fpr, tpr, thresholds = roc_curve(y_test, y_test_predprob, pos_label=1)

yd = max(tpr - fpr)

maxindex = (tpr - fpr).tolist().index(max(tpr - fpr))

y_test_preds = [1 if i >= thresholds[maxindex] else 0 for i in y_test_predprob]

cm = confusion_matrix(y_test, y_test_preds)

TN, FP, FN, TP = confusion_matrix(y_test, y_test_preds).ravel()

# Overall accuracy

ACC = (TP+TN)/(TP+FP+FN+TN)

ACC = round(ACC, 4)

# Sensitivity, hit rate, recall, or true positive rate

TPR = TP/(TP+FN)

TPR = round(TPR, 4)

# Specificity or true negative rate

TNR = TN/(TN+FP)

TNR = round(TNR, 4)

# Precision or positive predictive value

PPV = TP/(TP+FP)

PPV = round(PPV, 4)

# Negative predictive value

NPV = TN/(TN+FN)

NPV = round(NPV, 4)

# Fall out or false positive rate

FPR = FP/(FP+TN)

FPR = round(FPR, 4)

# False negative rate

FNR = FN/(TP+FN)

FNR = round(FNR, 4)

# False discovery rate

FDR = FP/(TP+FP)

FDR = round(FDR, 4)

# 准确率

acc = accuracy_score(y_test, y_test_preds)

acc = round(acc, 4)

# 精确率

pre = precision_score(y_test, y_test_preds)

pre = round(pre, 4)

# 召回率

rec = recall_score(y_test, y_test_preds)

rec = round(rec, 4)

# 特异度

spe = (y_test[y_test == y_test_preds] == 0).sum()/(y_test == 0).sum()

spe = round(spe, 4)

# f1分数

f1 = f1_score(y_test, y_test_preds)

f1 = round(f1, 4)

# auc

roauc = roc_auc_score(y_test, y_test_predprob)

roauc = round(roauc, 4)

if output:

print('Accuracy: {0:.4f}'.format(acc))

print('Precision: {0:.4f}'.format(pre))

print('Recall: {0:.4f}'.format(rec))

print('f1_score: {0:.4f}'.format(f1))

print('AUC: {0:.4f}'.format(auc))

res = {}

res['model'] = clf

res['acc'] = acc

res['pre'] = pre

res['rec'] = rec

res['f1'] = f1

res['roauc'] = roauc

res['yd'] = yd

res['y_test_predprob'] = y_test_predprob

res['fpr'] = fpr

res['tpr'] = tpr

res['params'] = clf.get_params()

res['spe'] = spe

res['cm'] = cm

res['ACC'] = ACC

res['TPR'] = TPR

res['TNR'] = TNR

res['PPV'] = PPV

res['NPV'] = NPV

return res

def evalu_models(self, names, sampling_methods, X_test, y_test, save=False):

acc_dict = {}

pre_dict = {}

rec_dict = {}

spe_dict = {}

f1_dict = {}

roauc_dict = {}

yd_dict = {}

auc_std_dict = {}

auc_ci_dict = {}

params_dict = {}

pvalue_dict = {}

cm_dict = {}

aucprint_dict = {}

ACC_dict = {}

TPR_dict = {}

TNR_dict = {}

PPV_dict = {}

NPV_dict = {}

for (name, method) in zip(names, sampling_methods):

if name == 'SVM':

open = True

else:

open = False

# acc, pre, rec, f1, roauc, yd, y_test_predprob, fpr, tpr = self.cal_res(method, X_test, y_test, output=False)

res = self.cal_res(method, X_test, y_test, output=False, sm=open)

acc_dict[name] = res["acc"]

pre_dict[name] = res["pre"]

rec_dict[name] = res["rec"]

spe_dict[name] = res["spe"]

f1_dict[name] = res["f1"]

roauc_dict[name] = res["roauc"]

yd_dict[name] = res["yd"]

params_dict[name] = res['params']

cm_dict[name] = res['cm']

ACC_dict[name] = res['ACC']

TPR_dict[name] = res['TPR']

TNR_dict[name] = res['TNR']

PPV_dict[name] = res['PPV']

NPV_dict[name] = res['NPV']

acc_ser = pd.Series(acc_dict)

pre_ser = pd.Series(pre_dict)

rec_ser = pd.Series(rec_dict)

spe_ser = pd.Series(spe_dict)

f1_ser = pd.Series(f1_dict)

roauc_ser = pd.Series(roauc_dict)

yd_ser = pd.Series(yd_dict)

params_ser = pd.Series(params_dict)

cm_ser = pd.Series(cm_dict)

aucprint_ser = pd.Series(aucprint_dict)

ACC_ser = pd.Series(ACC_dict)

TPR_ser = pd.Series(TPR_dict)

TNR_ser = pd.Series(TNR_dict)

PPV_ser = pd.Series(PPV_dict)

NPV_ser = pd.Series(NPV_dict)

df = pd.DataFrame({

# 'params':params_ser,

'AUC':roauc_ser,

# 'AUC_95%CI':auc_ci_ser,

# 'AUC_pvalue':pvalue_ser,

'NPV':NPV_ser,

'PPV':PPV_ser,

'SENS':TPR_ser,

'SPEC':TNR_ser,

'F1 score':f1_ser

})

if save:

df.to_csv('evalu_model.csv', index=True)

return df

def miss_fillup(df, cols, method):

from sklearn.impute import SimpleImputer

for col in cols:

tmp = df.loc[:,col].values.reshape(-1,1)

imp = SimpleImputer(strategy=method).fit_transform(tmp)

df.loc[:,col] = imp

return df

def col_valcount(df, col):

return df[col].value_counts().sort_index()

dt = pd.read_excel("SR.xlsx")

cols = ['复发', '序号', '住院号', 'BMI', '年龄.1', '性别', '合并症（糖尿病/高血压）',

'肝炎 (0无 1乙肝 2丙肝）', '腹水', '直径(1,<5,5-10,2,3>10)', '数量.1',

'肝硬化', '肝内出血', '肝内坏死', '动脉周边强化', '肝内脂肪', '包膜完整', '肿瘤内动脉穿插',

'DSA造影动静脉瘘（0：无；1：有）', 'DSA造影动门脉瘘（0：无；1：有）', 'DSA造影弥漫染色（0：结节肿块型；1：弥漫型）',

'DSA造影侧枝循环（0：无；1：有）', '碘油沉积良好', 'Unnamed: 28',

'MVI', '卫星灶', 'TKI', 'PD1', 'ALB.1', 'ALT.1', 'AST.1', 'TBIL.1',

'ALBI.1', 'PT.1', 'INR.1', 'PLT.1', 'CRP.1', 'Cre.1', 'Neu.1', 'Ly.1', 'BCLC分期 ',

'AFP.1']

dt = dt[cols]

cols_name = {

'年龄.1':'Age',

'性别':'Gender',

'合并症（糖尿病/高血压）':'Comorbidity',

'肝炎 (0无 1乙肝 2丙肝）':'Etiology',

'腹水':'Ascites',

'直径(1,<5,5-10,2,3>10)':'Tumor size',

'数量.1':'Tumor number',

'肝硬化':'Liver cirrhosis',

'肝内出血':'Intrahepatic hemorrhage',

'肝内坏死':'Intratumoral necrosis',

'动脉周边强化':'PAE',

'肝内脂肪':'Intratumoral fat',

'包膜完整':'Complete capsule',

'肿瘤内动脉穿插':'Intratumoral arterial',

'DSA造影动静脉瘘（0：无；1：有）':'AVF',

'DSA造影动门脉瘘（0：无；1：有）':'APVF',

'DSA造影弥漫染色（0：结节肿块型；1：弥漫型）':'DDS',

'DSA造影侧枝循环（0：无；1：有）':'CCG',

'碘油沉积良好':'GIOD',

'Unnamed: 28': 'OR',

'卫星灶':'Satellite lesion',

'ALB.1':'ALB',

'ALT.1':'ALT',

1. **Supplementary Tables**

**Supplementary Table 1. Systemic downstaging therapy regimens.**

| **Regimens** | **Patient No (%)** |
| --- | --- |
| *Tyrosine Kinase Inhibitors* | |
| Sorafenib | 36 (7.4) |
| Lenvatinib | 49 (10) |
| Apatinib | 31(6.4) |
| Donafenib | 11(2.3) |
| Apatinib | 12 (2.3) |
| Regorafenib | 22 (4.7) |
| *Immune Checkpoint Inhibitors* | |
| Toripalimab | 45 (9.2) |
| Camrelizumab | 42 (8.6) |
| Tislelizumab | 20 (4.1) |
| Sintilimab | 7 (1.4) |

**Supplementary Table 2. Variables Selected Using XGBoost and the Corresponding Variable Importance Score.**

| **Variables** | **Importance Score** |
| --- | --- |
| Microvascular invasion (present vs. absent) | 23.221 |
| Objective response to downstaging therapy (no vs. yes) | 15.892 |
| α-fetoprotein (>400 vs. ≤ 400 ng/ml) | 9.834 |
| Intratumoral artery (present vs. absent) | 9.436 |
| Age (>65 vs. ≤ 65 years) | 8.182 |
| Neutrophils (>1.5 vs. ≤ 1.5 ng/ml) | 7.490 |
| Tumor number (>three vs. ≤three) | 6.672 |
| Diffuse distribution of staining (present vs. absent) | 6.011 |
| TACE combined with tyrosine kinase inhibitors (yes vs. no) | 5.582 |
| The Albumin-Bilirubin grade (2-3 vs. 1) | 5.290 |
| Alanine aminotransferase (U/L) | 4.528 |
| Necrosis or severe ischemia (present vs. absent) | 4.283 |

| **Supplementary Table 3. Parameters of the XGBoost model.** | |
| --- | --- |
| **Parameters** | |
| learning_rate | 0.02 |
| n_estimators | 191 |
| max_depth | 3 |
| min_child_weight | 1 |
| gamma | 0.1 |
| subsample | 0.85 |
| colsample_bytree | 0.85 |
| scale_pos_weight | 1.24 |
| reg_alpha | 1 |
| reg_lambda | 4 |
| seed | 324 |

**3. Supplementary Figures**


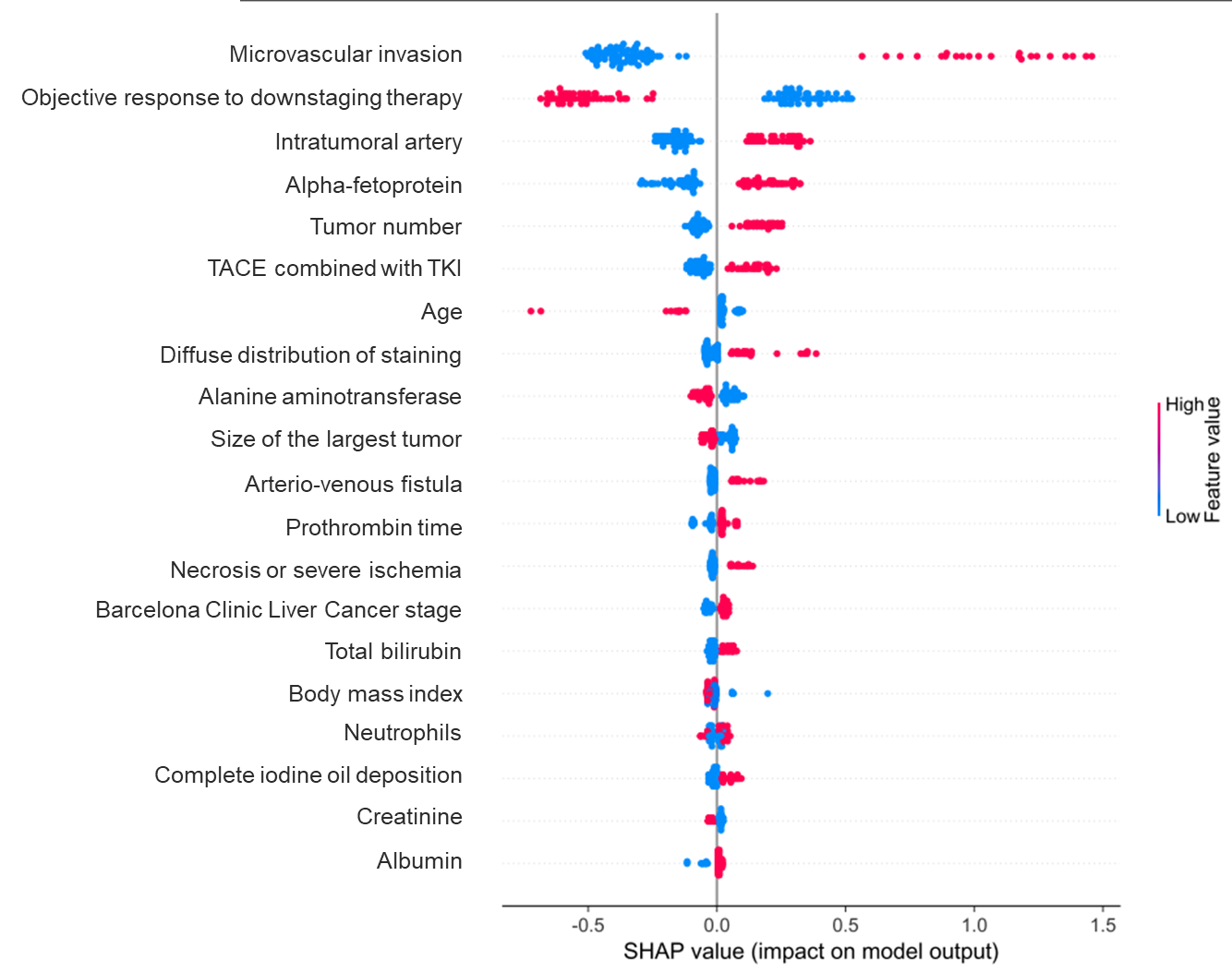


**Supplementary Figure 1.** The most important variables rank in XGBoost model.

Abbreviations: TACE = transarterial chemoembolization; TKI = tyrosine kinase inhibitor.


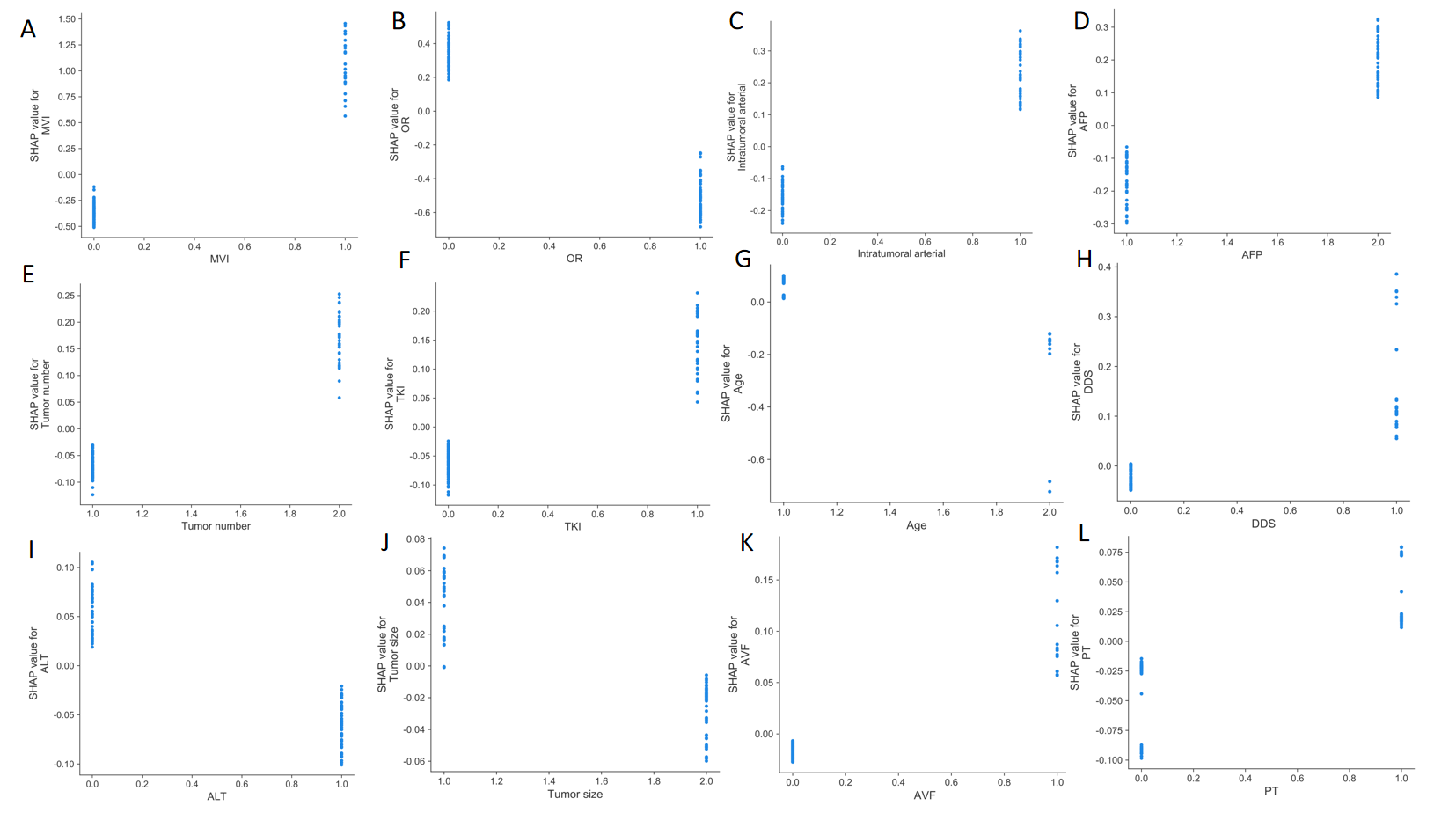


**Supplementary Figure 2.** SHAP explain the nonlinear impact of the indicator variables on disease-free survival.
